# Crop yield prediction integrating genotype and weather variables using deep learning

**DOI:** 10.1371/journal.pone.0252402

**Published:** 2021-06-17

**Authors:** Johnathon Shook, Tryambak Gangopadhyay, Linjiang Wu, Baskar Ganapathysubramanian, Soumik Sarkar, Asheesh K. Singh

**Affiliations:** 1 Department of Agronomy, Iowa State University, Ames, IA, United States of America; 2 Department of Mechanical Engineering, Iowa State University, Ames, IA, United States of America; World Energy and Meteorology Council, UNITED KINGDOM

## Abstract

Accurate prediction of crop yield supported by scientific and domain-relevant insights, is useful to improve agricultural breeding, provide monitoring across diverse climatic conditions and thereby protect against climatic challenges to crop production. We used performance records from Uniform Soybean Tests (UST) in North America to build a Long Short Term Memory (LSTM)—Recurrent Neural Network based model that leveraged pedigree relatedness measures along with weekly weather parameters to dissect and predict genotype response in multiple-environments. Our proposed models outperformed other competing machine learning models such as Support Vector Regression with Radial Basis Function kernel (SVR-RBF), least absolute shrinkage and selection operator (LASSO) regression and the data-driven USDA model for yield prediction. Additionally, for providing interpretability of the important time-windows in the growing season, we developed a temporal attention mechanism for LSTM models. The outputs of such interpretable models could provide valuable insights to plant breeders.

## Introduction

One of the key challenges in plant breeding and crop production is to predict performance (for example, seed yield) in unseen and new environments. This active research area is complicated by the time and expense of generating an extensive dataset to represent a wide range of genotypes and environments. Among different crops, soybean has a long history of cultivation in North America, with the first reported production in Georgia in 1766 [1]. Over the years, production in the US and Canada has expanded longitudinally as far west as Kansas-Colorado border and latitudinally from southern Texas to Canada [2, 3]. North American annual soybean yield trials (known as Uniform Soybean Tests (UST)) have been coordinated in the United States and Canada through the United States Department of Agriculture (USDA) between public breeders in university and government settings since 1941 [4, 5]. These trials are used to evaluate current and experimental varieties in multiple environments within their range of adaptation. Therefore, these trials are valuable sources of historical and current data to improve prediction performance with the assimilation of genetic and environmental variables.

Management and permanent environmental effects have been examined primarily at small scales due to the labor required for managing large numbers of plots [6, 7]. With the addition of each layer of added characterization of the environment, less of the differences need be ascribed to a generic “environmental” component, and can instead be examined individually in combination with plant genetics. The nexus of genetic and non-genetic variables form the cornerstone of plant breeding strategies, irrespective of crop species, for meeting crop production challenges in the future [8, 9].

Climatic resiliency in cultivars is an important objective for plant breeders and farmers to get a high seed yield in a myriad of environments [10]. The climatic variability can be associated with changes in temperature and rainfall events (including patterns and magnitude) and other weather variables. In addition to spatial variability, temporal variability of weather variables [11] is equally important but generally less understood in yield prediction studies. It is important to understand how agricultural production is affected by the variability of weather parameters in the presence of global climate change, especially with higher occurrence of extreme weather events. Therefore, prediction of the effects of changing environments on performance can help in making informed plant breeding decisions, marketing decisions, optimizing production and comparing results over multiple years [12].

Traditionally, crop growth models have been proposed to simulate and predict crop production in different scenarios including climate, genotype, soil properties, and management factors [13]. These provide a reasonable explanation on biophysical mechanisms and responses but have deficiencies related to input parameter estimation and prediction in complex and unforeseen circumstances [14]. Previous attempts at yield prediction across environments have relied on crop models generated by quantifying response in a limited number of lines while altering a single environmental variable, limiting the inference scope [15]. To bypass the limitations of crop growth models, linear models have also been used to predict yield with some success [16]. However, these low-capacity models typically rely on a rather small subset of factors, therefore failing to capture the complexity of biological interactions and more site-specific weather variable complexities. Traditional linear methods such as Autoregressive Integrated Moving Average (ARIMA) have been used for time series forecasting problems [17], but these methods are effective in predicting future steps in the same time-series. Considering the importance of climate extremes for agricultural predictions, random forest has been utilized to predict grid-cell deviations of yields [18]. For time series prediction tasks, deep neural networks show robustness to noisy inputs and also have the capability to approximate arbitrary non-linear functions [19]. Deep learning models can provide solutions in the presence of such complex data comprising of different weather variables, maturity groups and zones, and genotype information. These models can be highly efficient in learning the non-linear dependencies between multivariate input data (weather variables along with maturity group, cluster information) and the predicted yield.

Long Short Term Memory (LSTM) networks are very useful for time series modeling as they can capture the long-term temporal dependencies in complex multivariate sequences [20]. LSTMs have shown state-of-the-art results in various applications including off-line handwriting recognition [21], natural language processing [22] and engineering systems [23]. LSTMs have also been used effectively for multivariate time series prediction tasks [24–26]. LSTM based model has been used for corn yield estimation [27], but these models lack interpretability as well. is based on geospatial data without field-scale farming management data and lacks temporal resolution in the absence of daily weather data.

In recent years, a significant number of different approaches has been proposed to address the ‘interpretability’ problem [28–33]. The use of ‘attention” mechanism is one such approach where the goal is to localize important parts of input features by using the attention weights. Attention-based model [34] was originally introduced for neural machine translation to outperform the encoder-decoder model [22, 35]. While use of ‘attention’ has been predominant in the computer vision community [36], the domain of sequence modeling, time-series modeling has extensively used this concept recently [37–39]. Additionally, it is becoming increasingly evident to the ML community that the attention mechanism in time-series modeling can not only help in prediction performance, it can also be used for interpretability [25, 40–44].

Attention based LSTM has been used along with multi-task learning (MTL) output layers [45] for county level corn yield anomaly prediction only based on meteorological data (maximum daily temperature, minimum daily temperature) without field-scale farming data. Previous work [46] using deep learning for yield prediction has utilized multi-spectral images to predict yield (instead of leveraging only multivariate time series as input) without considering model interpretability. Khaki et al. [47] applied deep neural networks for yield prediction of maize hybrids using environmental data, but their model is not capable of explicitly capturing the temporal correlations and also lacks interpretability. Other approaches to predict yield rely on the use of sensors to identify the most informative set of variables to predict yield [48, 49], which is very useful in multiple applications. However, there is still a need to integrate weather parameters using a time series approach involving multiple genotypes. In addition to accurate prediction, the ability to interpret domain-relevant prediction outcomes from a machine learning model (learning temporal dependencies from multivariate time series) can significantly benefit the domain experts in the field of plant breeding.

Using these motivations, we developed a model that can capture the temporal variability of different weather variables across the growing season in an interpretable manner to predict soybean yield from the UST dataset of field trials spanning 13 years across 28 U.S. states and Canadian provinces. We propose a framework based on LSTM and temporal attention to predict crop yield with 30 weeks (spanning the typical crop growing season) of weather data per year (over 13 years) provided as input, along with a reduced representation of the pedigree to capture differences in the response of varieties to the environment. We varied the number of input time-steps and compared the performance of our proposed Temporal Attention model with the Stacked LSTM model for two variations of each model. We also compared against the results of SVR-RBF, LASSO regression and the data-driven state-of-the-art USDA model [16]. The temporal attention mechanism highlights the significant time periods during the growing season leading to high or low yield prediction. The results from the temporal attention are in concord with the domain knowledge. In this paper, we report improved fidelity interpretation of the prediction outcomes without sacrificing the accuracy for multivariate time-series prediction. Our proposed framework can have widespread applications in plant breeding, crop science research, and agricultural production.

## Material and methods

### Preparation of performance records

Files from 2003–2015 USTs were downloaded as PDFs [4, 5]. Using on-line utility Zamzar (zamzar.com), all 26 PDFs from this period were converted to.xlsx files, with each tab corresponding to a single page in the file. In this way, the vast majority of tables were recovered with no errors or need for human translation. However, random checking for error was manually performed to ensure verity. These tables were manually curated to align all performance records for a given genotype/location combination into a single row. Records that did not have yield data (due to a variety not being planted in a specific location or dying prior to production of seed), were removed from the file.

**Fig 1:**
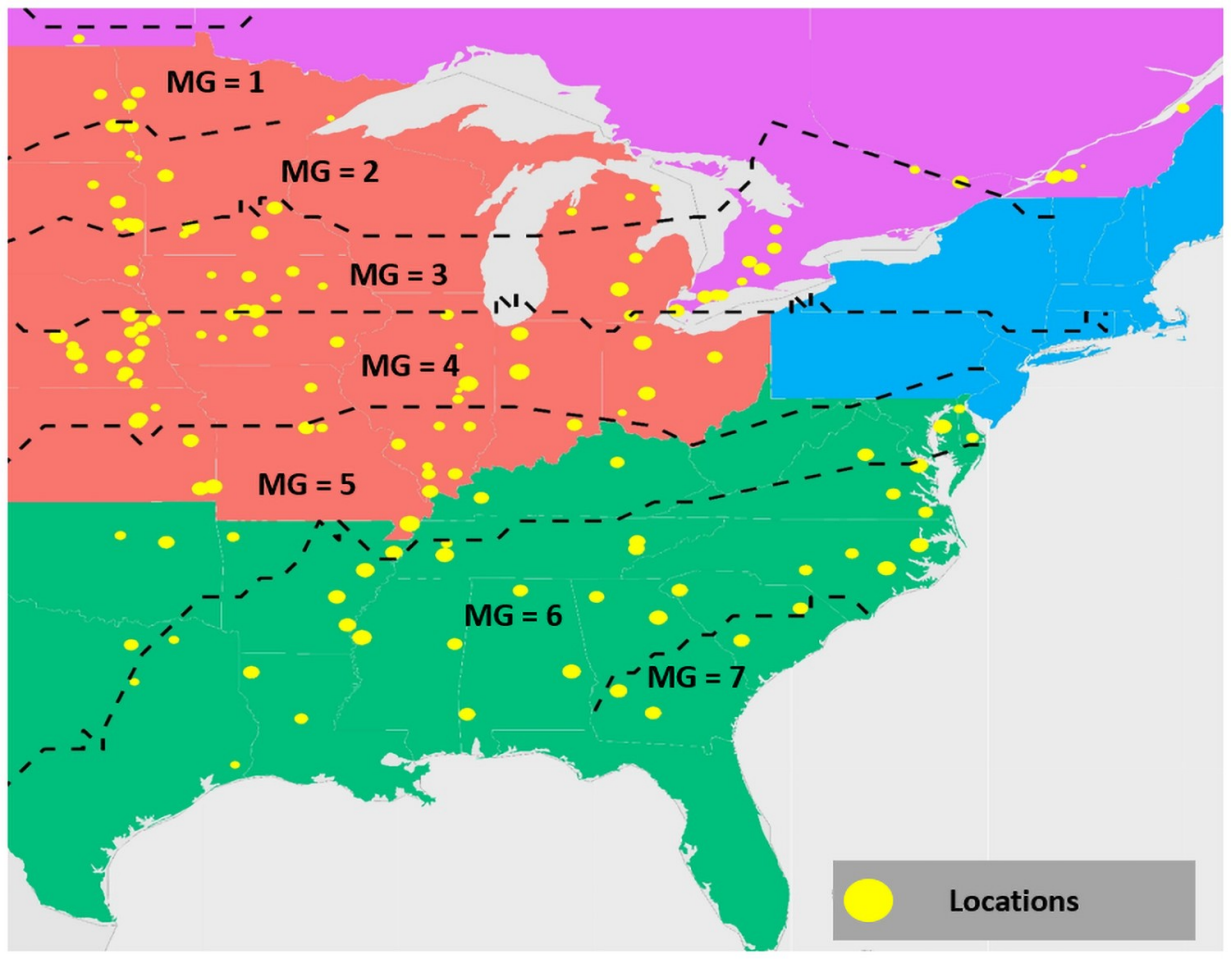
Map showing different locations in the USA and Canada included in our dataset. The dataset comprises of different maturity groups (MGs), some of which are labeled in the figure. The relative size of a yellow dot (representing location) indicates the size of the dataset for that particular location. Dataset included observations from the National Uniform Soybean Tests for years 2003–2015 and is split into North (MG 0 to 4) and South (MG 4 to 8) regions [50, 51], consisting of 103,365 performance records over 13 years and 150 locations. These records are matched to weekly weather data for each location throughout the growing season (30 weeks). This generated a dataset with 35,000 plots having phenotype data for all agronomic traits.

**Fig 2:**
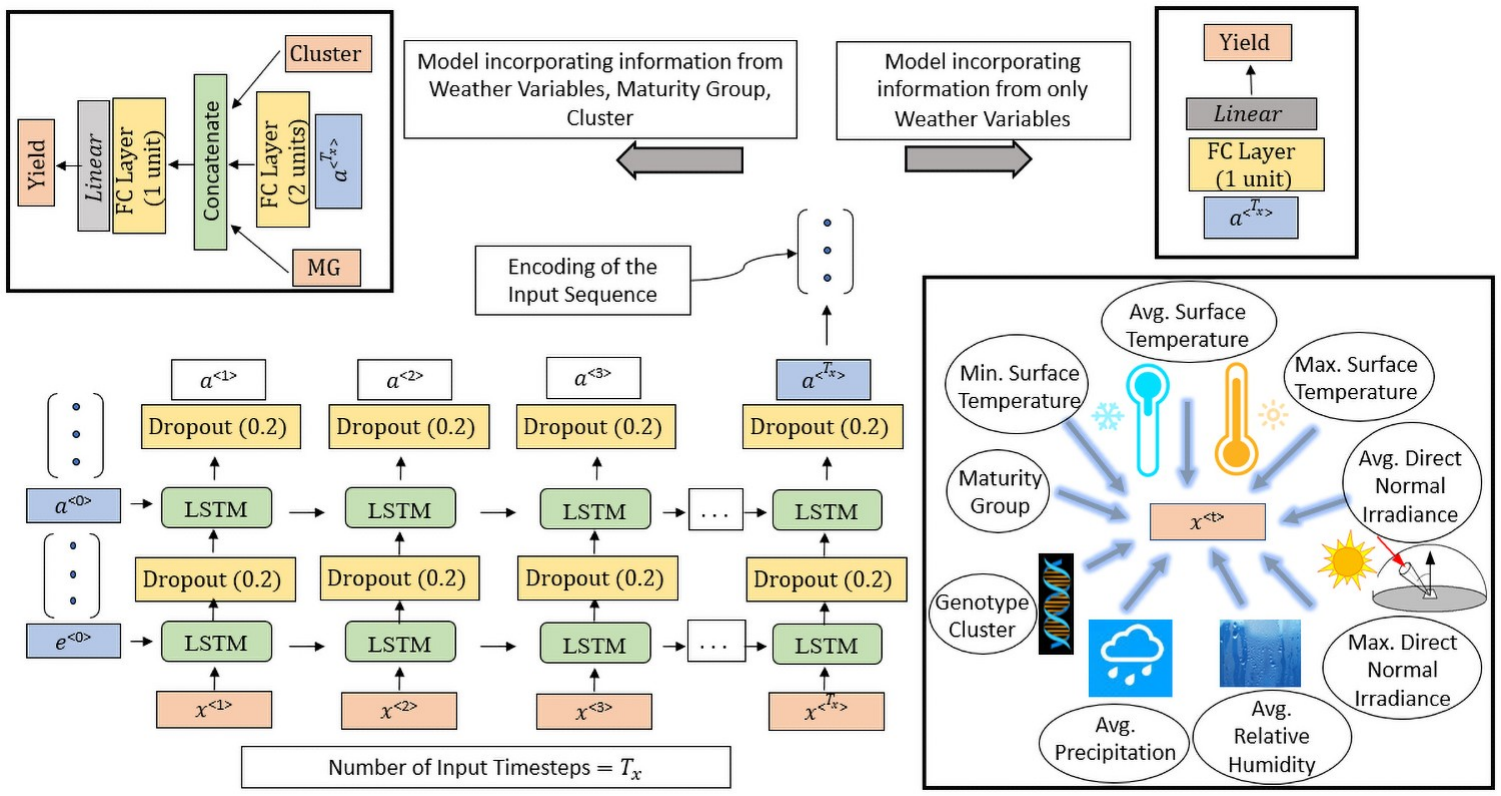
Stacked LSTM model. The input feature vector is *x*^<*t*>^ at time-step ‘t’. Depending on whether the MG and genotype cluster information are incorporated in the model or not, the vector *x*^<*t*>^ can be 9-dimensional or 7-dimensional. We included 7 weather variables in our study. The embedding vector *a*^<*T*_*x*_>^ encodes the entire input sequence and summarizes the sequential dependencies from the time-step 0 to the time-step *T*_*x*_. We designed two variants of our proposed model based on input information with the time series encoding part remaining the same for both variants. This model (when including MG, cluster with *T*_*x*_ = 30) had 202,503 learnable parameters and the training time/epoch was 18 secs.

Following data cleaning, the final dataset comprised of 103,365 performance records over 13 years representing 5839 unique genotypes. The map in Fig 1 shows the different locations. After compilation, we imported performance records in Python for further data analysis. Each performance record comprises of 214 days (crop growing season, defined April 1 through October 31) of multivariate time-series data and each day is represented by 7 weather variables as shown in Fig 2.

### Acquisition and sub-sampling of weather records

Daily weather records for all location/year combinations were compiled based on the nearest grid point from a gridded 30km product (Weather.com [52]). We downsampled the dataset to include maximum, minimum, and average conditions on different time frames throughout the growing season (defined April 1 through October 31) and this information was appended to performance records. Additional details regarding this are provided in S5 Text.

### Genotype clustering

We included genotype-specific criteria to apply the model for specific genotypes and mean location yield across genotypes. Due to the nature of the UST program, most of the genotypes tested in this period do not have molecular marker data available, preventing the use of a G matrix. To circumvent these restrictions, we developed a completely connected pedigree for all lines with available parentage information, resulting in the formation of a 5839 x 5839 correlation matrix. To improve the model performance, genotypes were clustered based on the organization which developed them, providing additional control over relatedness.

We find that the optimum number of clusters for the K-means algorithm can be considered as 20 (details provided in S1 Text). We clustered genotypes in 20 clusters using the K-means Clustering technique based on the correlation matrix to extract information about relatedness. With a specified number of clusters (*n*), the K-means algorithm finds *n* groups of equal variance by choosing centroids of the clusters to minimize a criterion known as *inertia* (also called, within-cluster sum-of-squares). This algorithm is effective for a large number of samples and finds application across different domains [53–55]. With this hard clustering technique, each genotype belongs to one of the 20 clusters. The cluster ID for a genotype is augmented into the proposed model for yield prediction.

### Model development

A modeling approach based on recurrent neural network (RNN) can capture correlation across time. Long short-term memory (LSTM) [56] can overcome the error backflow problems of an RNN [57]. By learning long-range correlations in a sequence, LSTM can accurately model complex multivariate sequences [20].

**Fig 3:**
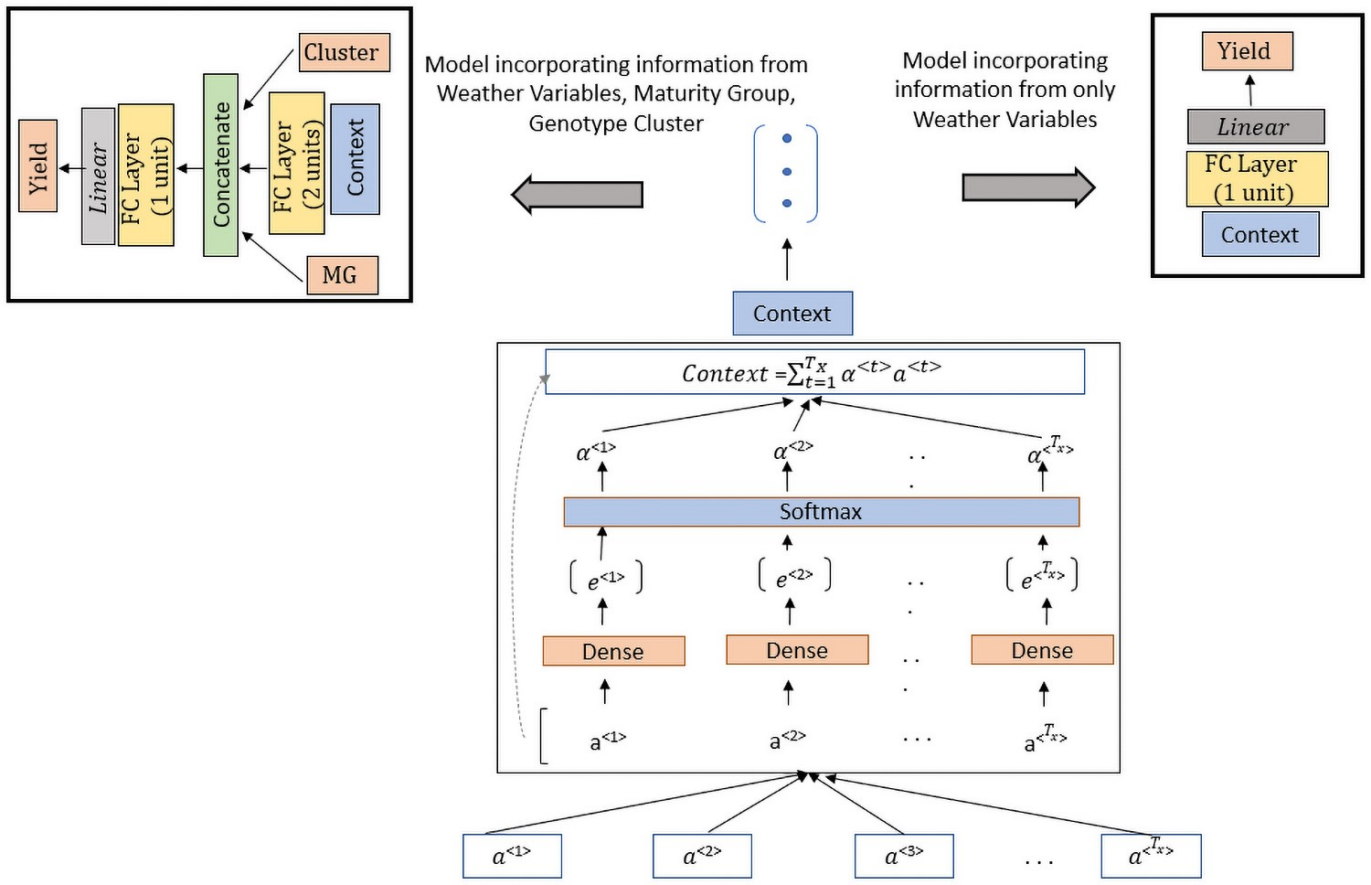
Temporal attention model. The LSTM encoding part is the same as that of the Stacked LSTM Model where we get the annotations *a*^<*t*>^ for each timestep. Instead of only using *a*^<*T*_*x*_>^, this model utilizes all annotations which act as inputs for the temporal attention mechanism. Based on the computed context vector, the two variants of this model are designed depending on the input information. This model (when including MG, cluster with *T*_*x*_ = 30) had 202,632 learnable parameters and the training time/epoch was 18 secs.

We developed two models, based on LSTM: (a) Stacked LSTM Model (without using any attention) (Fig 2), and (b) Temporal Attention Model (using a temporal attention mechanism) (Fig 3). The output of both the models is yearly seed yield as this is a many-to-one prediction problem. For each model, we formulated the model variants depending on whether the performance records comprise data of maturity group (MG) and genotype cluster. The same modeling approach was used to compute the time-step wise encoding for both models. Two stacked LSTM layers were used to encode the *T*_*x*_ time-steps of the input sequence as shown in Fig 2. Depending on the variant, for both models, we concatenated MG and genotype cluster values with the compressed time-series information.

In the Stacked LSTM Model, the last hidden state of the encoding part is assumed to be the compressed representation from the entire input sequence. This fixed-dimensional representation was concatenated with maturity group and cluster information before the prediction of yield (Fig 2). For the Temporal Attention Model, the compressed information (context) is computed after aggregating the information from the sequence of hidden states using the attention mechanism. The concept of soft temporal attention [34] was first proposed in the context of neural machine translation to overcome the bottleneck of the encoder-decoder model [22, 35] for long sequences. Compressing all information from the input time-steps into a fixed-length single vector was the major bottleneck for the encoder-decoder model. Temporal attention can be applied to many-to-many time series prediction [25] and many-to-one-prediction [44, 58]. The proposed approach (Fig 3) does not incorporate a decoder LSTM as we are performing a many-to-one prediction problem. Taking in the annotations of all time-steps as input, the attention block aggregates the information and computes the context vector. Therefore, while the Stacked LSTM model only considers the hidden state of the last encoding time-step, the Temporal Attention model considers the hidden state of all the time-steps across the crop growing season and learns the alignments in order to compute the aggregated context.

A greedy search method was utilized to empirically determine the most influential weather variable on seed yield prediction considering data of both the northern and southern U.S. regions. In the first step of the greedy search, the Stacked LSTM model was trained for each of the 7 variables and to choose the variable that had the least RMSE. With this variable added, in the second step, the model was trained for each of the other 6 variables. In this way, variables were added.

All input features were scaled in the range (-1, 1) with the scaler fitted on the training set. Data were randomly split into training (80%), validation (10%) and test (10%) sets. To get the best set of hyper-parameters, we performed several experiments. For training the models, Adam optimizer was used [59] (learning rate of 0.001 gave better results) and the mean squared error loss function was computed. After each LSTM layer, dropout layer (0.2) was used to prevent overfitting. The hidden state dimensions of the two LSTM layers were kept same for simplicity. Hidden State dimension of 128 showed better performance in our experiments. The reduction in dimension of the time-series encoding (context) was optimized to 2 units. Both models were trained for 100 epochs with batch size of 512 to get the optimal error scores. Models were developed using Keras [60] with the TensorFlow backend [61] and the models were trained using one NVIDIA Titan RTX GPU. For both the Stacked LSTM and Temporal Attention model, the training time per epoch is 6s.

We utilized three evaluation metrics: root mean square error (RMSE), mean absolute error (MAE), and coefficient of determination or R-squared (*R*^2^) score. We computed the metrics after inverting the applied scaling to have forecasts and the actual values in the original scale. For comparison of the empirical results, we used two baseline models: Support Vector Regression with Radial Basis Function kernel (SVR-RBF) and least absolute shrinkage and selection operator (LASSO) regression. We also tried to optimize the hyper-parameters of the baseline models by running multiple experiments. The SVR-RBF and LASSO models are developed using the scikit-learn [62] library. For the SVR-RBF model, in our experiments, we find that the optimal value of epsilon is 0.1 with the regularization parameter (C) kept as 1. The LASSO is a linear model trained with L1 prior as regularizer. Alpha is the constant that multiplies the L1 term. With the maximum number of iterations kept as 1000, through different experiments, we optimize alpha to 0.000001. The results for SVR-RBF and LASSO model are given in S2 and S3 Tables respectively.

## Results

### Greedy search

#### With the inclusion of only weather variables

We observed average relative humidity had the lowest test RMSE. With the inclusion of average relative humidity in the prediction model, average direct normal irradiance was the next most important variable. Sequentially, the remaining weather variables (from S4 Table) were: maximum direct normal irradiance, maximum surface temperature, minimum surface temperature, average surface temperature, and average precipitation.

#### With the inclusion of weather variables, MG and genotype clusters

Minimum surface temperature was the most important weather variable, i.e, had the lowest RMSE. The remaining weather variables (from S4 Table) with diminishing importance were—average direct normal irradiance, average surface temperature, maximum direct normal irradiance, average precipitation, average relative humidity, and maximum surface temperature. Noticeably, the ranking of the variables was different but the absolute change in MAE scores were minimal.

### Empirical results

For determination of appropriate temporal sampling of weather information to predict yield using our proposed frameworks, the validation set RMSE was used to determine optimal (lowest RMSE) number of time points to predict seed yield. Using a step-wise approach building from monthly, bi-weekly, weekly and finally daily data, almost similar comparative performance was observed in each scenario except for daily (Validation RMSE = 7.595). The intermediate scenario of weekly data was picked for some subsequent analyses, to facilitate faster training of LSTMs and also not to downsample to a higher extent in capturing the long-range temporal dependencies.

Overall, a *R*^2^ score of 0.796 between predicted and observed yields in the test set was attained; largely capturing the differences in performance between environments and years. However, the model remains somewhat limited in its ability to generate genotype-specific yield predictions due to the limited complexity of relationships which can be modeled using LSTM, and a lack of genomic information on each genotype. As currently implemented, the model’s mean absolute error is 5.441 bu/acre, which is reasonable given the levels of variability within a given environment/year combination. For example, in Ames, IA, during 2003, yields ranged from 33.3–55.3 bu/acre. In spite of this large range of difference, mean absolute error of only 4.57 bu/ac was observed for this environment. No perceptible trends are observed when we looked at state wide results combined over years. We also looked at originating breeding state as well as private company entries, and no geographical trends were noticeable. The comparative results are shown in Table 1.

**Table 1:** Comparison of performance of the two deep learning models (2 variants of each model based on the input information) with SVR-RBF and LASSO by varying the input sequence length (*T*_*x*_) using metrics of the test set. Each model was trained three times, to obtain the average and standard deviation of each evaluation metric.

*T*_*x*_	Model	Weather Variables			Including All		
		RMSE	MAE	*R*^2^ Score	RMSE	MAE	*R*^2^ Score
7	LASSO	14.465±0.000	11.485±0.000	0.184±0.000	14.463±0.000	11.485±0.000	0.185±0.000
	SVR-RBF	8.465±0.000	6.285±0.000	0.720±0.000	7.913±0.000	6.012±0.000	0.755±0.000
	Stacked LSTM	**8.290±0.021**	6.191±0.007	**0.731±0.001**	7.257±0.025	5.470±0.024	0.794±0.001
	Temporal Attention	8.292±0.018	**6.172±0.027**	**0.731±0.001**	**7.243±0.037**	**5.453±0.030**	**0.795±0.001**
15	LASSO	13.732±0.000	10.911±0.000	0.265±0.000	13.729±0.000	10.912±0.000	0.266±0.000
	SVR-RBF	8.438±0.000	6.256±0.000	0.722±0.000	7.835±0.000	5.943±0.000	0.760±0.000
	Stacked LSTM	**8.284±0.029**	**6.176±0.034**	**0.732±0.002**	7.248±0.020	5.460±0.015	0.795±0.001
	Temporal Attention	**8.284±0.017**	6.182±0.035	**0.732±0.001**	**7.226±0.017**	**5.441±0.013**	**0.796±0.001**
30	LASSO	12.811±0.000	9.995±0.000	0.360±0.000	12.790±0.000	9.987±0.000	0.363±0.000
	SVR-RBF	8.460±0.000	6.282±0.000	0.721±0.000	7.875±0.000	5.976±0.000	0.758±0.000
	Stacked LSTM	**8.283±0.005**	**6.178±0.004**	**0.732±0.000**	7.276±0.050	5.484±0.049	0.792±0.002
	Temporal Attention	8.303±0.019	6.200±0.013	0.730±0.001	**7.239±0.032**	**5.441±0.028**	**0.795±0.002**

**Fig 4:**
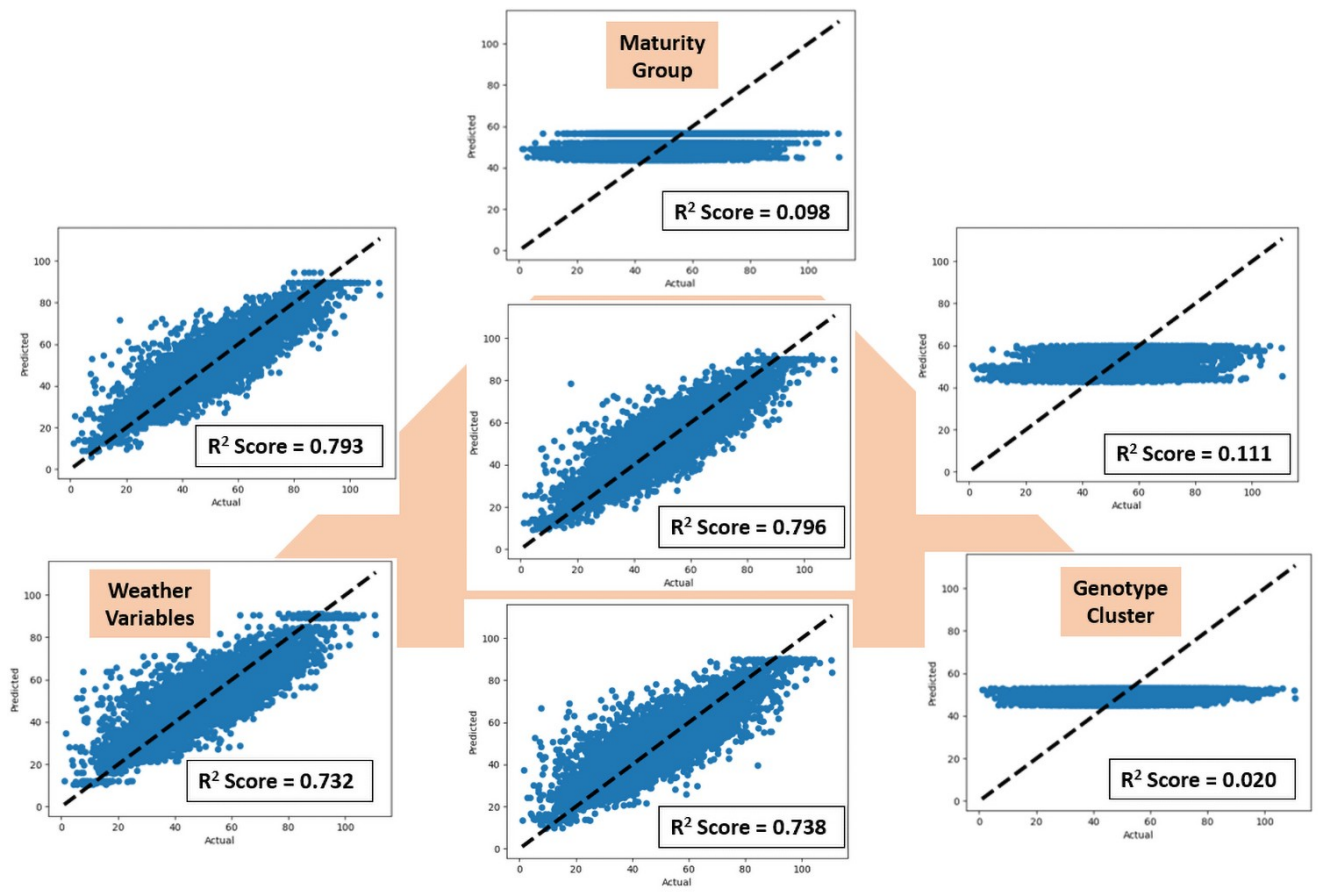
Results for different inputs to the temporal attention model. The vertices of the triangle demonstrate results including only the MG, only genotype cluster and only weather variables in the input. The edges show the results with a combination of inputs from the respective vertices. The results showed improvement when the genotype cluster was included with weather variables. The coefficient of determination increased further when MG was included with weather variables. The best results were noticed when information from all sources was incorporated (shown at the center of the triangle).

Varying the value of *T*_*x*_ did not have a significant change in the accuracy of the models, except for the LASSO model. Augmenting the information of maturity group and cluster to the weather variables (detailed description of the chosen approach given in S6 Text) seem to have a considerable impact in the performance of the deep learning models. Both of our proposed models (Stacked LSTM, Temporal Attention) showed similar performance, and results improved when more information were included (Fig 4). The coefficient of determination was highest (0.796) when information from all the sources (MG, genotype cluster, weather variables) were incorporated. The best performance (test MAE = 5.441) from Temporal Attention model is 10.72% of the average seed yield for the test set (50.745) and 33.96% of the standard deviation of the test set (16.019)(Fig 4). Comparatively, test MAE of 9.987 was obtained from LASSO, while SVR-RBF test MAE was 5.976 with same input features. Both the Stacked LSTM and the Temporal Attention models outperformed LASSO and SVR-RBF models.

In comparison with the data-driven state-of-the-art USDA model [16], our deep learning LSTM approach performs significantly better, evident from much lower absolute errors. The USDA approach uses a linear regression approach with coefficients based on historical statewide yields and weather averages. However, the USDA model does not predict performance for individual locations. Ths USDA model averages over the entire state, while our model was built on predictions from multiple separate locations within a state. The USDA model is a state (farm level) model, while our model is on a breeding level scale (finer scale). The results from the USDA model are outputted as one single crop yield, while our model outputs multiple different yields from each plot and per location. Due to this limitation of the USDA model, we compared results of our model with the USDA model using year wise average across states for the test set. In comparison with the USDA model, the absolute errors of the Temporal Attention model are lower for most of the 13 years (except in 2004, 2011 and 2013). For 2014 and 2015, the absolute errors of deep learning models were 0.38 and 0.17 (compared to 1.32 and 1.70 for the USDA model), respectively. Detailed comparison results are provided in the (S1 Table).

### Interpretability

**Fig 5:**
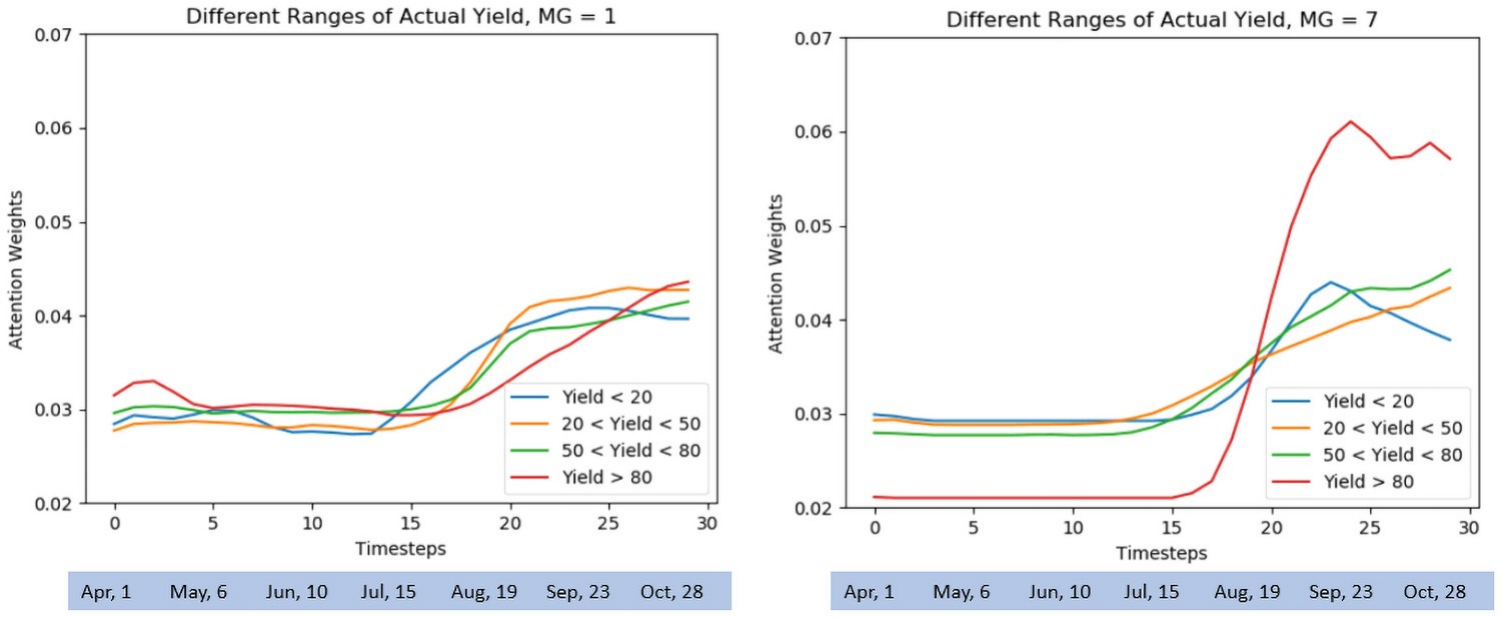
Distribution of attention weights for the entire input sequence (spanning the growing season). Considering different ranges of actual yield, the results are demonstrated for two different maturity groups (MG = 1, MG = 7) providing stark geo-climatic regions (Fig 1). Early season variables were observed to be comparatively less important for prediction of the highest yielding genotypes.

In addition to accurate yield prediction, the Temporal Attention Model provided insights (Fig 5) about how early-season variables were less important for yield prediction in the highest yielding genotypes for two geographically distinct maturity groups: MG1 (Northern US adaptation) and MG7 (Southern US adaptation). We observed mild sigmoid curves for the highest yielding group in the case of both MG1 and MG7. However, we note that while MG1 had a significantly large number of plots (≈550) for the highest yielding group, MG7 had only about 30 such plots. It points to the increasing importance of features in the August–September time phases for both these North and South US regions. These time phases coincide with pod set and seed fill stages, emphasizing their importance in the final yield, and need functional validation which is outside of the scope of our research. However, these insights are a useful as a hypotheses generation tool and another advantage of these models motivating future research.

## Discussion

We establish the potential for use of a long short-term memory-based method for yield prediction to allow models to account for temporal differences in the occurrence of weather events. Predictions using this system can be made reasonably accurate due to a large amount of training data made available through the mining of historical records. Our approach (using LSTM and attention) is an efficient modeling scheme to analyze soybean crop growth interaction with the weather, as well as to identify key physio-environmental features that are important to include into any predictive model. For example, differences in the timing of extreme heat events, as well as drought periods, would affect soybean plants in various ways depending on the stage of plant development. For example, heat stress during flowering is particularly damaging while heat in vegetative stages of development may have less drastic impact on seed yield [63]. With a larger encompassing dataset, breeders and researchers can be empowered to parse out most informative time periods, weather variables and crop responses. This information sets up the framework for breeding strategies to develop climate resilient and responsive varieties.

### Relative importance of weather variables

Our results show a potential mismatch in the heuristic and empirical results for the importance of weather variables in predicting seed yield. The finding of minimum surface temperature as the most significant weather variable suggests that nighttime temperatures play a larger role in yield prediction than previously suggested [64]. Our study is a retrospective design, and cannot conclude definitively that this is the case; however, these findings necessitate further empirical investigations and can be used to formulate the next set of hypotheses. This finding is significant, as minimum temperatures have been reported to be increasing at a faster rate than maximum temperatures [65]. More studies are needed to ascertain the relative importance of these variables and can motivate morpho-physiological attentive breeding approaches to assemble sturdier varieties for future scenarios. While greedy search is a very useful tool as demonstrated in our approach, in future we envision a more scalable attention-based approach (similar to the temporal attention concept we present), to provide additional insights about the importance of variables in predictions. This approach will be computationally less intensive, but more importantly it will better integrate spatial importance of variables more intrinsically compared to the brute greedy search.

### Advantages of our framework

Our work shows a unique strategy to assimilate and utilize complex data for seed yield prediction. For comparison purposes, we compared our models with the SVR-RBF, LASSO and the data-driven USDA model. The USDA model has a limitation on the type of data it can utilize and is limited in its application. For example, as the USDA model computes predictions at the state level, the finer resolution available with our model may help in making regional marketing decisions, as well as in creating yield predictions which can capture intra-state variation due to factors such as differences in rainfall (amount, duration, time) in different areas of the state. Since our results are built on more than a decade of data, it also reflects that early season weather variables are less useful in seed yield prediction and needs empirical evidence to confirm the genetic variability in plasticity of soybean genotypes in earlier stages of growth and development. Importantly, we emphasize that the utilization of the attention module within a LSTM framework allows us to tease out potentially important features for further testing. This alleviates the disadvantage of DL models—which serve as purely blackbox predictive models—by allowing for hypothesis generation that will allow scientific insight via targeted follow up analysis and experiments.

The advantages of LSTM based models have been recently established for maize yield prediction at a county level [27], but the model lacked interpretability. Attention based LSTM along with multi-task learning (MTL) output layers has also been used for maize yield prediction using county level data based on meteorological data (maximum daily temperature, minimum daily temperature, and daily precipitation) [45]. These studies are important for solving the yield prediction challenge; however, models are based on geospatial data without field-scale farming management data and variety information is indiscernible, and based on limited weather variables.

### Future expansion and generalizability of our framework

A large capacity machine learning approach, such as the one presented in this paper using LSTM and temporal attention will be robust to incorporate weather changes and adjust performance predictions accordingly. Additional information that may improve the results of this approach is the inclusion of any supplemental irrigation provided, soil fertility levels, disease pressure and resistance levels, and direct genetic markers for the tested varieties, all of which would further strengthen predictive ability. While a lack of molecular marker data for each line precludes us from leveraging genomic prediction and integrating with the LSTM model, this is a logical next step as such data becomes available. The models performed reasonably well in absence of crop management and soil data, but these can be incorporated in future studies to further leverage the predictive ability of our models. Therefore, future implementations may be expanded to include genomic data, additional factors such as preceding crop, row spacing, planting date, soil texture, or additional temporal data in the forms of soil sensor measurements and remote sensing data for morphological and physiological traits. The approach presented in this work will further enhance phenomics assisted breeding and prescriptive breeding that use in-season data from different sensors and payloads [48, 49, 66] using machine and deep learning approaches suitable in plant sciences applications [67–69]. The basic framework of LSTM for the phenotypic prediction can be applied to any crop and trait with weather-dependent variability in order to better understand the genotype x environment effects found in the course of multi-environment testing. While we present the usefulness of LSTM and attention based models on seed yield predictions and associated variable importance, this approach is trait agnostic and will be useful for a wide variety of useful traits including root traits [70, 71].

### Broad societal impact

The ability to make accurate predictions of crop performance can lead to optimization across many different levels of organizations. At the federal level, improved crop insurance recommendations can be made based on weather forecasts before planting, and be continually updated throughout the season as more data is recorded and forecasts are updated. Crop insurance decisions for providers and clients rely on weather events. This is reflected, for example, in the date of planting and risk associated with seedling frost damage. The same is applicable for fall season killing frost. Therefore, both insurers and farmers can benefit from a robust crop performance prediction that includes weather parameters. These losses are not just from weather related events such as frost, but also drought, and diseases/insect-pest that may also be indirectly related to weather conditions. Furthermore, individual and area plans can benefit from crop yield prediction; and yield as well as revenue protection type of crop insurance will be impacted from better crop performance prediction built on weather. Railroads, grain cooperatives, and end-users can streamline the logistics of handling the desired quantities of grain if they are permitted a better understanding of how much grain (and of what quality) will be produced in a given region. Farmers can make better marketing decisions if they have an accurate and high confidence prediction of their production for the year, allowing them to sell their crops at the most opportune time. For example, farmers can better utilize price guarantees or price locks in the futures market if they have better prediction tools at their disposal. We envision that similar work on other crops and over a longer time span will generate invaluable insights for cultivar development and plant breeding and production related research in a challenging climate.

## Conclusion

Unraveling the importance of different weather parameters would be a substantial step forward in understanding the impact of climate change on variety’s plasticity. Viewed through the lens of interpretability, DL based predictive models vs process based predictive models have distinct pros and cons. Process based models have clear relationships (by construction); however the dependency is limited to the confines of the model parameters, and it is non-trivial to assimilate additional data to extract broader trends. On the other hand, accurately interpreting DL based model outcomes is an open problem in the AI/ML community, with much activity. However, DL models (in contrast with the process-based models) have the ability to seamlessly assimilate additional data. Our vision is therefore to evaluate if systematically augmenting DL based predictive models with increasing amounts of physio-morphological informative features provides a way towards unraveling scientific insights. We can accomplish this by deploying our DL framework as a ‘hypotheses generation tool’. We build DL models using a large volume of data and variety of information incorporating domain based knowledge. We can then systematically probe the impact of various physio-morphological and environmental parameters on yield (via sensitivity analysis, and “what if” scenario evaluation), and establish a framework to generate hypotheses in different crop species and physio-morphological characteristics under different climatic conditions. Until explicitly interpretable DL becomes feasible, the hypotheses generation DL models will have the maximum impact in meeting the need of climate change scenarios and to incorporate plasticity response in future varieties.

## Supporting information

S1 Fig Clustering details. After finding the optimum number of clusters (20), we plot magnitude against cardinality.
(TIF)

S2 Fig LSTM block. The input, output and forget gates regulate whether information can be augmented or removed from the cell state [72, 73].
(PDF)

S3 Fig Encoding of input sequence. LSTM is used for encoding the input sequence which is of length *T*_*x*_ and the output from the first LSTM layer is a batch of sequences that are propagated through another layer of LSTM. We used dropout regularization after each LSTM layer to prevent overfitting.
(TIF)

S4 Fig Temporal attention mechanism. The context vector is computed and the attention weights are learned simultaneously.
(PDF)

S5 Fig Data availability. Attempt to gain insights behind performance on the test set based on data availability in the training set.
(TIF)

S1 Table Comparative performance with the USDA model. The deep learning model showed better performance.
(PDF)

S2 Table Performance of SVR-RBF model for different epsilon values. The optimal value of epsilon is found to be 0.1.
(PDF)

S3 Table Performance of LASSO model for different alpha values. The optimal value of alpha is found to be 0.000001.
(PDF)

S4 Table Greedy search for the weather variables. With inclusion of only weather variables and with inclusion of MG, genotype cluster and weather variables.
(PDF)

S1 Text Clustering. Clustering is an unsupervised machine learning technique used to group unlabeled examples. A metric (similarity measure) is used to estimate the similarity between examples by combining the examples’ feature data. With the increase in the number of features, the similarity measure computation can become more complex. By assigning a number to each cluster, each complex example is represented by a cluster-ID. This makes clustering a simple yet powerful technique that finds applications in domains including image segmentation, anomaly detection, social network analysis, and medical imaging. The output of the clustering technique (Cluster ID) can be then used as input instead of a high-dimensional feature for machine learning algorithms.
(PDF)

S2 Text Long Short Term Memory networks (LSTMs). Recurrent Neural Networks (RNNs) can explicitly capture temporal correlations in time series data, and efficient learning of the temporal dependencies leads to highly accurate prediction and forecasting, often outperforming static networks. Deep RNNs are trained using the error backpropagation algorithm; however, the propagation of error gradients through the latent layers and unrolled temporal layers suffer from the vanishing gradient problem. Therefore, gradient descent of an error criterion may be inadequate to train RNNs especially for tasks involving long-term dependencies [57]. Moreover, standard RNNs fail to learn in the presence of time lags greater than 5–10 discrete time-steps between relevant input events and target signals [72]. To overcome these challenges, Long short-term memory (LSTM) was used, which is an RNN architecture designed to overcome the error backflow problems [56]. By using input, output and forget gates to prevent the memory contents being perturbed by irrelevant inputs and outputs, LSTM networks have the ability in learning long-range correlations in a sequence and can accurately model complex multivariate sequences [20]. The cell state in an LSTM block can allow the information to just flow along with it unchanged and information can be added to or removed from the cell state. In an LSTM block (S2 Fig), there are input, output and forget gates that prevent the perturbation of the memory contents with irrelevant information. These gates regulate the augmentation of any information to the cell state.
(PDF)

S3 Text Encoding using stacked LSTM. To encode the information of the input time-steps, we had two LSTM layers stacked on top of each other to get the *T*_*x*_ annotations as shown in S2 Fig. We finalized this model after an extensive hyper-parameter and architecture search. The Stacked LSTM can capture the long-range dependencies and temporal correlations for nonlinear data, therefore, they were ideal for our research problem. An LSTM layer consists of a sequence of directed nodes where each node corresponds to a single time-step. The first layer of LSTM takes in input the information from all timesteps sequentially. For input at each time-step, we concatenated the information from different variables and this concatenated information acts as the input to the LSTM node. The LSTM node computes the hidden state as a function of the previous hidden state and the input vector for the current timestep. Each LSTM node updates the hidden state and cell state. The next LSTM node receives as input these updated states and the concatenated information of that time-step. After performing computations at each timestep of the time series, the first LSTM layer generates a sequence of hidden states for input to the next LSTM layer. The encodings returned by the first LSTM layer act as inputs for the next LSTM layer (S3 Fig).
(PDF)

S4 Text Temporal attention mechanism. The input to the temporal attention mechanism is a sequence of vectors and the aim is to compute aggregated information from these vectors. The vectors are annotations corresponding to the input time-steps. We computed the context vector from the weighted sum of annotations (hidden states) as shown in S4 Fig. Annotation *a*^<*t*>^ focuses on the information surrounding the time-step *t* in the sequence. The attention weight *α*^<*t*>^ signifies the contribution of the information at a time-step *t* for prediction.
(PDF)

S5 Text Downsampling the dataset. The original multivariate time-series data set comprises of 214 days (thus having 214 time-steps). Each day is represented by 7 weather variables. The variables are Average Direct Normal Irradiance (ADNI, *Wm*^−2^), Average Precipitation Previous Hour (AP, *inches*), Average Relative Humidity (ARH, *Percentage*), Maximum Direct Normal Irradiance (MDNI, *Wm*^−2^), Maximum Surface Temperature (MaxSur, °*C*), Minimum Surface Temperature (MinSur, °*C*) and Average Surface Temperature (AvgSur, °*C*). We consider the first 210 days (time-steps) while downsampling the daily data to weekly (*T*_*x*_ = 30), biweekly (*T*_*x*_ = 15) and monthly (*T*_*x*_ = 7) values. We kept the sense of the variables the same while downsampling and thus instead of considering mean for all the variables, we compute an average of average (ADNI, ARH, AvgSur), maximum of maximum (MDNI, MaxSur), minimum of minimum (MinSur). For average precipitation, we consider both the total precipitation and the average precipitation for the considered time interval (7 days, 14 days, 30 days). The model performs better when the downsampling is performed using average precipitation and therefore we implement this in our experiments.
(PDF)

S6 Text Adding maturity group, genotype cluster informations. We performed three different experiments to optimize the augmentation of the information of Maturity Group (MG). To do this, we developed three different architectures. In the first approach, we concatenated MG to every time-step of the input. In the second approach, we concatenated MG to every time-step and also after the 2nd LSTM layer just before prediction. For the third architecture, we added MG only after the 2nd LSTM. The second approach which gave the least RMSE became our selected approach for the paper. Interestingly, we observe that even though MG is a static value for all time-steps, approach 1 gave better results than approach 3. With MG information already augmented, we performed a similar search to select a suitable architecture for adding the genotype cluster information. We observed that approach 2 also gives the least RMSE in this case.
(PDF)

S7 Text Comparison with USDA model. We compare the performance of our deep learning model (Stacked LSTM) with the USDA’s weather-based soybean yield prediction model [16]. The USDA model which uses a linear regression approach doesn’t predict performance for individual locations. It predicts yield state-wise. Due to this limitation of the USDA model, we compare the models using year wise average across states for the test set (S1 Table). We computed the absolute error (between predicted and actual yield) for both the models. The deep learning model showed much-improved performance compared to the domain knowledge-based USDA model.
(PDF)

S8 Text Data availability—Insights. We try to gain insights based on data availability for performance on the test set as shown in S5 Fig. We plotted the test RMSE values using a heat map for all the (MG, Genotype Cluster) combinations. We also plotted the ratio (number of samples in training set/number of unique locations) to get an estimation of the data availability and data distribution for all the (MG, Genotype Cluster) combinations. From the figure, we observed that for the highest test RMSE (MG = 7, Cluster = 6) the corresponding data availability ratio is low. This holds true for most of the highest RMSE combinations, although not for all. Therefore, while not conclusive, it seems bad performance can be attributed to less data availability/location in the training set leading to high RMSE in the predicted yield. The framework developed in this paper allows similar investigations to motivate other insights, and future hypotheses driven research.
(PDF)
