# Supplementary figures and images for: Crop yield prediction integrating genotype and weather variables using deep learning
Source: PLoS One. 2021 Jun 17;16(6):e0252402. doi: 10.1371/journal.pone.0252402 (PMC8211294; doi:10.1371/journal.pone.0252402)

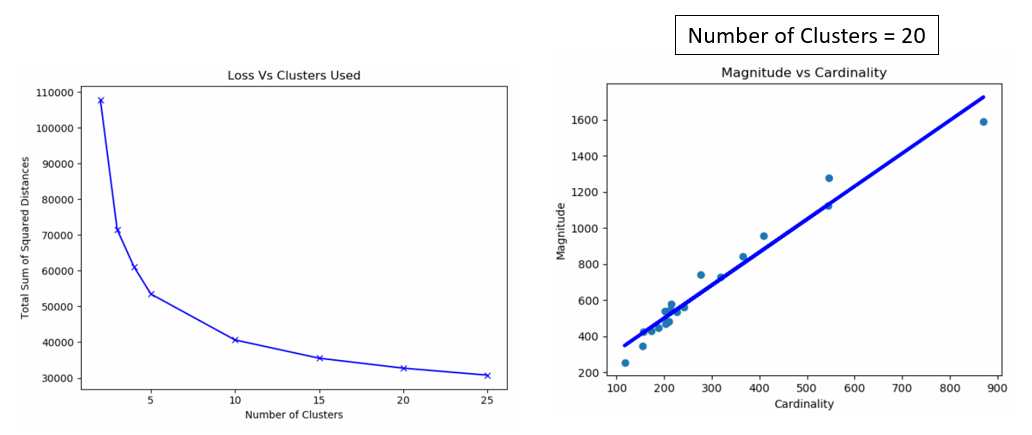

Supplement: S1 Fig — After finding the optimum number of clusters (20), we plot magnitude against cardinality. (TIF) [file pone.0252402.s001.tif]

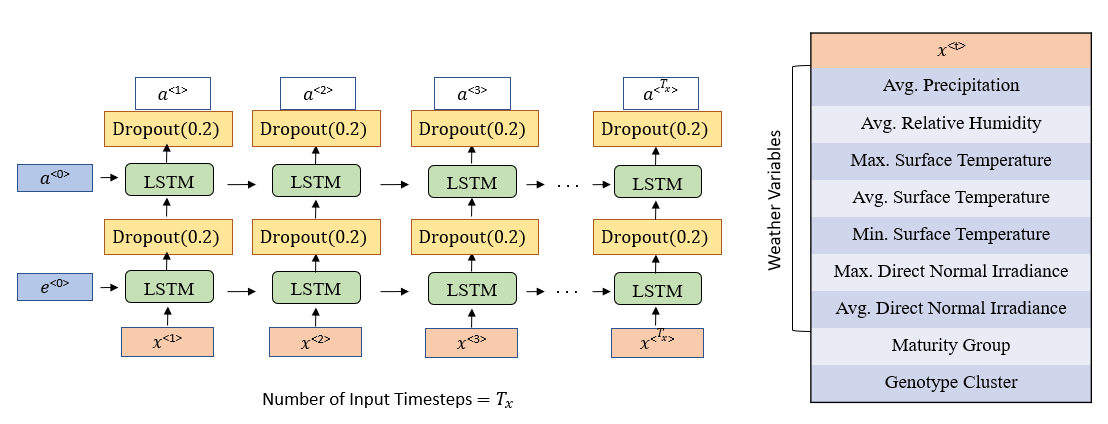

Supplement: S3 Fig — LSTM is used for encoding the input sequence which is of length Tx and the output from the first LSTM layer is a batch of sequences that are propagated through another layer of LSTM. We used dropout regularization after each LSTM layer to prevent overfitting. (TIF) [file pone.0252402.s003.tif]

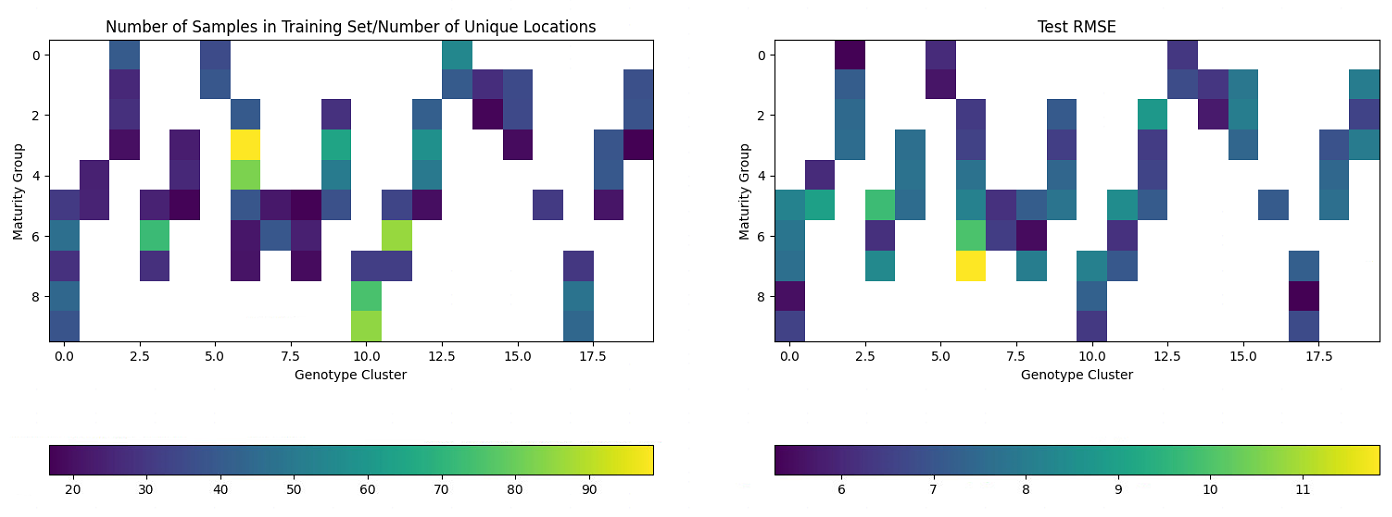

Supplement: S5 Fig — Attempt to gain insights behind performance on the test set based on data availability in the training set. (TIF) [file pone.0252402.s005.tif]
