# Supplementary material for: Crop yield prediction integrating genotype and weather variables using deep learning
Source: PLoS One. 2021 Jun 17;16(6):e0252402. doi: 10.1371/journal.pone.0252402 (PMC8211294; doi:10.1371/journal.pone.0252402)
Supplement: S2 Fig — The input, output and forget gates regulate whether information can be augmented or removed from the cell state [72, 73]. (PDF) [file pone.0252402.s002.pdf]

S2 Fig. LSTM block. The input, output and forget gates regulate whether information can be augmented or removed from the cell state.

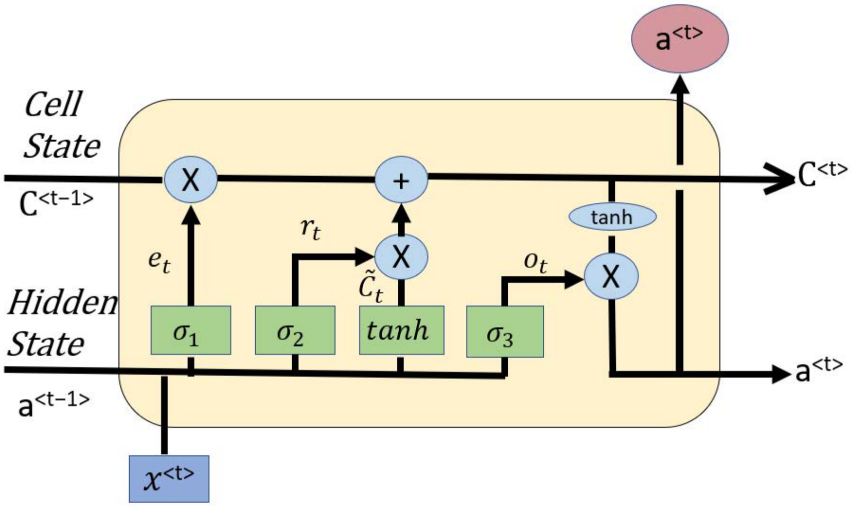

Forget gate layer is the first step of a LSTM block. A sigmoid layer (σ_1) decides the information to be removed the cell state of the previous time-step $C^{<t-1>}$. Forget gate naturally permits LSTM to learn local self-resets of memory contents that have become irrelevant [72]. This is performed as:

$$e_t = \sigma_1(W_e \cdot [a^{<t-1>}, x^{<t>}] + b_e)$$

The next step is to augment the cell state with new information. A sigmoid layer(σ_2, the input gate layer) followed by a tanh layer generates potential new information $\tilde{C}^{<t>}$ for augmentation.

$$r_t = \sigma_2(W_r \cdot [a^{<t-1>}, x^{<t>}] + b_r)$$
$$\tilde{C}^{<t>} = tanh(W_C \cdot [a^{<t-1>}, x^{<t>}] + b_C)$$

Thereafter, the new cell state $C^{<t>}$ is obtained as follows:

$$C^{<t>} = e_t \times C^{<t-1>} + r_t \times \tilde{C}^{<t>}$$

The hidden state of the previous time-step $a^{<t-1>}$ is passed through the third sigmoid layer (σ_3) for information selection. After combining with the new cell state $C^{<t>}$ (filtered with tanh layer), the new hidden state $a^{<t>}$ is computed as:

$$o_t = \sigma_3(W_o \cdot [a^{<t-1>}, x^{<t>}] + b_o)$$
$$a^{<t>} = o_t \times tanh(C^{<t>})$$

After updating, the values of $a^{<t>}$ and $C^{<t>}$ are passed to the LSTM block of the next time-step. The forget gate and output activation function is the most critical components of the LSTM block and removing any of them can significantly impair performance [73].
