# Supplementary material for: Crop yield prediction integrating genotype and weather variables using deep learning
Source: PLoS One. 2021 Jun 17;16(6):e0252402. doi: 10.1371/journal.pone.0252402 (PMC8211294; doi:10.1371/journal.pone.0252402)
Supplement: S4 Fig — The context vector is computed and the attention weights are learned simultaneously. (PDF) [file pone.0252402.s004.pdf]

S4 Fig. Temporal Attention Mechanism. The context vector is computed and the attention weights are learned simultaneously.

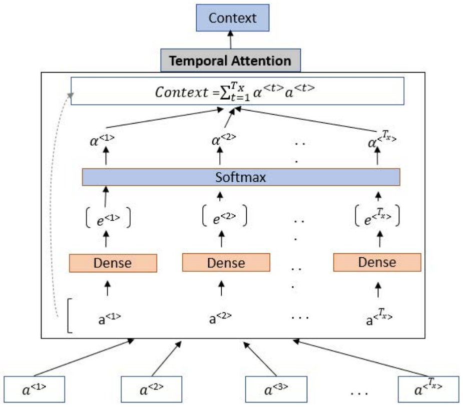

The context vector is computed like this:

$$context = \sum_{t=1}^{T_x} \alpha^{<t>} a^{<t>}$$

The attention weight for each annotation $a^{<t>}$ was computed using the softmax function.

$$\alpha^{<t>} = \frac{exp(e^{<t>})}{\sum_{t=1}^{T_x} exp(e^{<t>})}$$

The alignment model (dense layer, d) scores how well the input around time-step t is aligned with the prediction. It is parameterized as a feedforward neural network model as shown in S4 Fig. It is jointly trained with the entire network.

$$e^{<t>} = d(a^{<t>})$$
