# Supplementary material for: Crop yield prediction integrating genotype and weather variables using deep learning
Source: PLoS One. 2021 Jun 17;16(6):e0252402. doi: 10.1371/journal.pone.0252402 (PMC8211294; doi:10.1371/journal.pone.0252402)
Supplement: S1 Table — The deep learning model showed better performance. (PDF) [file pone.0252402.s006.pdf]

Year	Absolute Error (Deep Learning)	Absolute Error (USDA)
2003	0.30	5.49
2004	0.46	0.33
2005	0.13	2.75
2006	0.26	2.18
2007	0.09	0.37
2008	0.04	2.98
2009	0.09	0.84
2010	0.16	1.37
2011	0.25	0.06
2012	0.01	1.89
2013	0.69	0.27
2014	0.38	1.32
2015	0.17	1.70
