# Supplementary material for: Crop yield prediction integrating genotype and weather variables using deep learning
Source: PLoS One. 2021 Jun 17;16(6):e0252402. doi: 10.1371/journal.pone.0252402 (PMC8211294; doi:10.1371/journal.pone.0252402)
Supplement: S2 Table — The optimal value of epsilon is found to be 0.1. (PDF) [file pone.0252402.s007.pdf]

Epsilon	Validation RMSE
0.1	7.913
0.2	10.027
0.3	9.443
0.4	10.855
0.5	12.982
