# Supplementary material for: Crop yield prediction integrating genotype and weather variables using deep learning
Source: PLoS One. 2021 Jun 17;16(6):e0252402. doi: 10.1371/journal.pone.0252402 (PMC8211294; doi:10.1371/journal.pone.0252402)
Supplement: S3 Table — The optimal value of alpha is found to be 0.000001. (PDF) [file pone.0252402.s008.pdf]

**Alpha**	**Validation RMSE**
0.000001	12.763
0.00001	12.763
0.0001	12.821
0.001	13.745
0.01	15.772
0.1	15.994
1.0	15.994
