# Supplementary material for: Crop yield prediction integrating genotype and weather variables using deep learning
Source: PLoS One. 2021 Jun 17;16(6):e0252402. doi: 10.1371/journal.pone.0252402 (PMC8211294; doi:10.1371/journal.pone.0252402)
Supplement: S4 Table — With inclusion of only weather variables and with inclusion of MG, genotype cluster and weather variables. (PDF) [file pone.0252402.s009.pdf]

**Importance Order Of Weather Variables**	**With Only Weather Variables**	**With Weather Variables, Maturity Group and Genotype Clusters**
1 (With Lowest error)	Average Relative Humidity	Minimum Surface Temperature
2	Average Direct Normal Irradiance	Average Direct Normal Irradiance
3	Maximum Direct Normal Irradiance	Average Surface Temperature
4	Maximum Surface Temperature	Maximum Direct Normal Irradiance
5	Minimum Surface Temperature	Average Precipitation
6	Average Surface Temperature	Average Relative Humidity
7	Average Precipitation	Maximum Surface Temperature
