# Supplementary material for: Crop yield prediction integrating genotype and weather variables using deep learning
Source: PLoS One. 2021 Jun 17;16(6):e0252402. doi: 10.1371/journal.pone.0252402 (PMC8211294; doi:10.1371/journal.pone.0252402)
Supplement: S1 Text — Clustering is an unsupervised machine learning technique used to group unlabeled examples. A metric (similarity measure) is used to estimate the similarity between examples by combining the examples’ feature data. With the increase in the number of features, the similarity measure computation can become more complex. By assigning a number to each cluster, each complex example is represented by a cluster-ID. This makes clustering a simple yet powerful technique that finds applications in domains including image segmentation, anomaly detection, social network analysis, and medical imaging. The output of the clustering technique (Cluster ID) can be then used as input instead of a high-dimensional feature for machine learning algorithms. (PDF) [file pone.0252402.s010.pdf]

S1 Text. Clustering. Clustering is an unsupervised machine learning technique used to group unlabeled examples. A metric (similarity measure) is used to estimate the similarity between examples by combining the examples' feature data. With the increase in the number of features, the similarity measure computation can become more complex. By assigning a number to each cluster, each complex example is represented by a cluster-ID. This makes clustering a simple yet powerful technique that finds applications in domains including image segmentation, anomaly detection, social network analysis, and medical imaging. The output of the clustering technique (Cluster ID) can be then used as input instead of a high-dimensional feature for machine learning algorithms.

The choice of a clustering algorithm depends on whether it can scale efficiently to the available dataset. The clustering algorithms that compute the similarity between all pairs of examples, are not practical to be used for a large number of examples (n) as the runtime for this type of algorithm is proportional to the square of n. K-means clustering algorithm scales linearly with n and thus can be used for large-scale data. While the centroid-based clustering algorithm organizes data into non-hierarchical clusters, density-based clustering works by connecting highly dense areas into clusters. Density-based clustering is not suitable for data with high-dimensions and distribution-based clustering is not applicable when the data-distribution type is not known. Hierarchical clustering, which computes a tree of clusters is mostly meant for hierarchical data. This leads to the choice of a centroid-based algorithm for clustering the genotypes represented by a correlation matrix. K-means clustering is simple, efficient and the most commonly used centroid-based clustering algorithm. We implemented the K-means clustering algorithm in this work for these reasons.

We denote the set of examples as $\mathbf{X} = [\mathbf{x}_1, \mathbf{x}_2, ..., \mathbf{x}_n]$ and the set of cluster centres as $\mathbf{V} = [\mathbf{v}_1, \mathbf{v}_2, ..., \mathbf{v}_k]$. The first step of the K-means clustering algorithm is to randomly select 'k' number of cluster centres. In the first iteration, the distance between each example and cluster centre is computed. An example is assigned to a particular cluster for which the distance between the example and the cluster centre is minimum among all the cluster centres. For the i-th cluster, the new cluster centre is computed using:

$$\mathbf{v_i} = (1/c_i) \sum_{j=1}^{c_i} \mathbf{x}_j$$

where c_i is the number of examples in the i-th cluster. In the next iteration, the distance between each example and newly obtained cluster centre is computed. The assignment of examples to a cluster is performed based on the minimum distance. The iterations continue till no example is reassigned in a particular iteration.

Cluster cardinality denotes the number of examples per cluster. Cluster magnitude is the sum of distances from all examples to the centroid of the cluster. Intuitively, a higher cluster cardinality results in a higher cluster magnitude. Clusters are anomalous when cardinality doesn't correlate with magnitude relative to the other clusters. To find the optimum number of clusters, we run the K-means algorithm for increasing number of clusters. We compute the sum of cluster magnitudes and plot the total sum of squared distances against the number of clusters. The results are shown in S1 Fig. At $k = 20$, the reduction in loss becomes marginal with increasing 'k'. The optimal number of clusters is selected as 20. For $k = 25$, similar trend is obtained as that of $k = 20$, with higher cluster magnitude for higher cluster cardinality.
