# Supplementary material for: Crop yield prediction integrating genotype and weather variables using deep learning
Source: PLoS One. 2021 Jun 17;16(6):e0252402. doi: 10.1371/journal.pone.0252402 (PMC8211294; doi:10.1371/journal.pone.0252402)
Supplement: S2 Text — Recurrent Neural Networks (RNNs) can explicitly capture temporal correlations in time series data, and efficient learning of the temporal dependencies leads to highly accurate prediction and forecasting, often outperforming static networks. Deep RNNs are trained using the error backpropagation algorithm; however, the propagation of error gradients through the latent layers and unrolled temporal layers suffer from the vanishing gradient problem. Therefore, gradient descent of an error criterion may be inadequate to train RNNs especially for tasks involving long-term dependencies [57]. Moreover, standard RNNs fail to learn in the presence of time lags greater than 5–10 discrete time-steps between relevant input events and target signals [72]. To overcome these challenges, Long short-term memory (LSTM) was used, which is an RNN architecture designed to overcome the error backflow problems [56]. By using input, output and forget gates to prevent the memory contents being perturbed by irrelevant inputs and outputs, LSTM networks have the ability in learning long-range correlations in a sequence and can accurately model complex multivariate sequences [20]. The cell state in an LSTM block can allow the information to just flow along with it unchanged and information can be added to or removed from the cell state. In an LSTM block (S2 Fig), there are input, output and forget gates that prevent the perturbation of the memory contents with irrelevant information. These gates regulate the augmentation of any information to the cell state. (PDF) [file pone.0252402.s011.pdf]

S2 Text. Long Short Term Memory Networks (LSTMs). Recurrent Neural Networks (RNNs) can explicitly capture temporal correlations in time series data, and efficient learning of the temporal dependencies leads to highly accurate prediction and forecasting, often outperforming static networks. Deep RNNs are trained using the error backpropagation algorithm; however, the propagation of error gradients through the latent layers and unrolled temporal layers suffer from the vanishing gradient problem. Therefore, gradient descent of an error criterion may be inadequate to train RNNs especially for tasks involving long-term dependencies [57]. Moreover, standard RNNs fail to learn in the presence of time lags greater than 5-10 discrete time-steps between relevant input events and target signals [72]. To overcome these challenges, Long short-term memory (LSTM) was used, which is an RNN architecture designed to overcome the error backflow problems [56]. By using input, output and forget gates to prevent the memory contents being perturbed by irrelevant inputs and outputs, LSTM networks have the ability in learning long-range correlations in a sequence and can accurately model complex multivariate sequences [20]. The cell state in an LSTM block can allow the information to just flow along with it unchanged and information can be added to or removed from the cell state. In an LSTM block (S2 Fig), there are input, output and forget gates that prevent the perturbation of the memory contents with irrelevant information. These gates regulate the augmentation of any information to the cell state.
