# Supplementary material for: Crop yield prediction integrating genotype and weather variables using deep learning
Source: PLoS One. 2021 Jun 17;16(6):e0252402. doi: 10.1371/journal.pone.0252402 (PMC8211294; doi:10.1371/journal.pone.0252402)
Supplement: S3 Text — To encode the information of the input time-steps, we had two LSTM layers stacked on top of each other to get the Tx annotations as shown in S2 Fig. We finalized this model after an extensive hyper-parameter and architecture search. The Stacked LSTM can capture the long-range dependencies and temporal correlations for nonlinear data, therefore, they were ideal for our research problem. An LSTM layer consists of a sequence of directed nodes where each node corresponds to a single time-step. The first layer of LSTM takes in input the information from all timesteps sequentially. For input at each time-step, we concatenated the information from different variables and this concatenated information acts as the input to the LSTM node. The LSTM node computes the hidden state as a function of the previous hidden state and the input vector for the current timestep. Each LSTM node updates the hidden state and cell state. The next LSTM node receives as input these updated states and the concatenated information of that time-step. After performing computations at each timestep of the time series, the first LSTM layer generates a sequence of hidden states for input to the next LSTM layer. The encodings returned by the first LSTM layer act as inputs for the next LSTM layer (S3 Fig). (PDF) [file pone.0252402.s012.pdf]

S3 Text. Encoding Using Stacked LSTM. To encode the information of the input time-steps, we had two LSTM layers stacked on top of each other to get the T_x annotations as shown in S2 Fig. We finalized this model after an extensive hyper-parameter and architecture search. The Stacked LSTM can capture the long-range dependencies and temporal correlations for nonlinear data, therefore, they were ideal for our research problem. An LSTM layer consists of a sequence of directed nodes where each node corresponds to a single time-step. The first layer of LSTM takes in input the information from all timesteps sequentially. For input at each time-step, we concatenated the information from different variables and this concatenated information acts as the input to the LSTM node. The LSTM node computes the hidden state as a function of the previous hidden state and the input vector for the current timestep. Each LSTM node updates the hidden state and cell state. The next LSTM node receives as input these updated states and the concatenated information of that time-step. After performing computations at each timestep of the time series, the first LSTM layer generates a sequence of hidden states for input to the next LSTM layer. The encodings returned by the first LSTM layer act as inputs for the next LSTM layer (S3 Fig).
