# Supplementary material for: Crop yield prediction integrating genotype and weather variables using deep learning
Source: PLoS One. 2021 Jun 17;16(6):e0252402. doi: 10.1371/journal.pone.0252402 (PMC8211294; doi:10.1371/journal.pone.0252402)
Supplement: S4 Text — The input to the temporal attention mechanism is a sequence of vectors and the aim is to compute aggregated information from these vectors. The vectors are annotations corresponding to the input time-steps. We computed the context vector from the weighted sum of annotations (hidden states) as shown in S4 Fig. Annotation a focuses on the information surrounding the time-step t in the sequence. The attention weight α signifies the contribution of the information at a time-step t for prediction. (PDF) [file pone.0252402.s013.pdf]

S4 Text. Temporal Attention Mechanism. The input to the temporal attention mechanism is a sequence of vectors and the aim is to compute aggregated information from these vectors. The vectors are annotations corresponding to the input time-steps. We computed the context vector from the weighted sum of annotations (hidden states) as shown in S4 Fig. Annotation $a^{<t>}$ focuses on the information surrounding the time-step t in the sequence. The attention weight $\alpha^{<t>}$ signifies the contribution of the information at a time-step t for prediction.
