# Supplementary material for: Crop yield prediction integrating genotype and weather variables using deep learning
Source: PLoS One. 2021 Jun 17;16(6):e0252402. doi: 10.1371/journal.pone.0252402 (PMC8211294; doi:10.1371/journal.pone.0252402)
Supplement: S5 Text — The original multivariate time-series data set comprises of 214 days (thus having 214 time-steps). Each day is represented by 7 weather variables. The variables are Average Direct Normal Irradiance (ADNI, Wm−2), Average Precipitation Previous Hour (AP, inches), Average Relative Humidity (ARH, Percentage), Maximum Direct Normal Irradiance (MDNI, Wm−2), Maximum Surface Temperature (MaxSur, °C), Minimum Surface Temperature (MinSur, °C) and Average Surface Temperature (AvgSur, °C). We consider the first 210 days (time-steps) while downsampling the daily data to weekly (Tx = 30), biweekly (Tx = 15) and monthly (Tx = 7) values. We kept the sense of the variables the same while downsampling and thus instead of considering mean for all the variables, we compute an average of average (ADNI, ARH, AvgSur), maximum of maximum (MDNI, MaxSur), minimum of minimum (MinSur). For average precipitation, we consider both the total precipitation and the average precipitation for the considered time interval (7 days, 14 days, 30 days). The model performs better when the downsampling is performed using average precipitation and therefore we implement this in our experiments. (PDF) [file pone.0252402.s014.pdf]

S5 Text. Downsampling the dataset. The original multivariate time-series data set comprises of 214 days (thus having 214 time-steps). Each day is represented by 7 weather variables. The variables are Average Direct Normal Irradiance (ADNI, Wm^{-2}), Average Precipitation Previous Hour (AP, *inches*), Average Relative Humidity (ARH, *Percentage*), Maximum Direct Normal Irradiance (MDNI, Wm^{-2}), Maximum Surface Temperature (MaxSur, $^{\circ}C$), Minimum Surface Temperature (MinSur, $^{\circ}C$) and Average Surface Temperature (AvgSur, $^{\circ}C$). We consider the first 210 days (time-steps) while downsampling the daily data to weekly ($T_x = 30$), biweekly ($T_x = 15$) and monthly ($T_x = 7$) values. We kept the sense of the variables the same while downsampling and thus instead of considering mean for all the variables, we compute an average of average (ADNI, ARH, AvgSur), maximum of maximum (MDNI, MaxSur), minimum of minimum (MinSur). For average precipitation, we consider both the total precipitation and the average precipitation for the considered time interval (7 days, 14 days, 30 days). The model performs better when the downsampling is performed using average precipitation and therefore we implement this in our experiments.
