# Supplementary material for: Crop yield prediction integrating genotype and weather variables using deep learning
Source: PLoS One. 2021 Jun 17;16(6):e0252402. doi: 10.1371/journal.pone.0252402 (PMC8211294; doi:10.1371/journal.pone.0252402)
Supplement: S6 Text — We performed three different experiments to optimize the augmentation of the information of Maturity Group (MG). To do this, we developed three different architectures. In the first approach, we concatenated MG to every time-step of the input. In the second approach, we concatenated MG to every time-step and also after the 2nd LSTM layer just before prediction. For the third architecture, we added MG only after the 2nd LSTM. The second approach which gave the least RMSE became our selected approach for the paper. Interestingly, we observe that even though MG is a static value for all time-steps, approach 1 gave better results than approach 3. With MG information already augmented, we performed a similar search to select a suitable architecture for adding the genotype cluster information. We observed that approach 2 also gives the least RMSE in this case. (PDF) [file pone.0252402.s015.pdf]

S6 Text. Adding Maturity Group, Genotype Cluster Informations. We performed three different experiments to optimize the augmentation of the information of Maturity Group (MG). To do this, we developed three different architectures. In the first approach, we concatenated MG to every time-step of the input. In the second approach, we concatenated MG to every time-step and also after the 2nd LSTM layer just before prediction. For the third architecture, we added MG only after the 2nd LSTM. The second approach which gave the least RMSE became our selected approach for the paper. Interestingly, we observe that even though MG is a static value for all time-steps, approach 1 gave better results than approach 3. With MG information already augmented, we performed a similar search to select a suitable architecture for adding the genotype cluster information. We observed that approach 2 also gives the least RMSE in this case.
