# Supplementary material for: Crop yield prediction integrating genotype and weather variables using deep learning
Source: PLoS One. 2021 Jun 17;16(6):e0252402. doi: 10.1371/journal.pone.0252402 (PMC8211294; doi:10.1371/journal.pone.0252402)
Supplement: S7 Text — We compare the performance of our deep learning model (Stacked LSTM) with the USDA’s weather-based soybean yield prediction model [16]. The USDA model which uses a linear regression approach doesn’t predict performance for individual locations. It predicts yield state-wise. Due to this limitation of the USDA model, we compare the models using year wise average across states for the test set (S1 Table). We computed the absolute error (between predicted and actual yield) for both the models. The deep learning model showed much-improved performance compared to the domain knowledge-based USDA model. (PDF) [file pone.0252402.s016.pdf]

S7 Text. **Comparison with USDA Model.** We compare the performance of our deep learning model (Stacked LSTM) with the USDA's weather-based soybean yield prediction model [16]. The USDA model which uses a linear regression approach doesn't predict performance for individual locations. It predicts yield state-wise. Due to this limitation of the USDA model, we compare the models using year wise average across states for the test set (S1 Table). We computed the absolute error (between predicted and actual yield) for both the models. The deep learning model showed much-improved performance compared to the domain knowledge-based USDA model.
