# Supplementary material for: Crop yield prediction integrating genotype and weather variables using deep learning
Source: PLoS One. 2021 Jun 17;16(6):e0252402. doi: 10.1371/journal.pone.0252402 (PMC8211294; doi:10.1371/journal.pone.0252402)
Supplement: S8 Text — We try to gain insights based on data availability for performance on the test set as shown in S5 Fig. We plotted the test RMSE values using a heat map for all the (MG, Genotype Cluster) combinations. We also plotted the ratio (number of samples in training set/number of unique locations) to get an estimation of the data availability and data distribution for all the (MG, Genotype Cluster) combinations. From the figure, we observed that for the highest test RMSE (MG = 7, Cluster = 6) the corresponding data availability ratio is low. This holds true for most of the highest RMSE combinations, although not for all. Therefore, while not conclusive, it seems bad performance can be attributed to less data availability/location in the training set leading to high RMSE in the predicted yield. The framework developed in this paper allows similar investigations to motivate other insights, and future hypotheses driven research. (PDF) [file pone.0252402.s017.pdf]

S8 Text. Data Availability - Insights. We try to gain insights based on data availability for performance on the test set as shown in S5 Fig. We plotted the test RMSE values using a heat map for all the (MG, Genotype Cluster) combinations. We also plotted the ratio (number of samples in training set/number of unique locations) to get an estimation of the data availability and data distribution for all the (MG, Genotype Cluster) combinations. From the figure, we observed that for the highest test RMSE (MG = 7, Cluster = 6) the corresponding data availability ratio is low. This holds true for most of the highest RMSE combinations, although not for all. Therefore, while not conclusive, it seems bad performance can be attributed to less data availability/location in the training set leading to high RMSE in the predicted yield. The framework developed in this paper allows similar investigations to motivate other insights, and future hypotheses driven research.
